# Emodin Ameliorates Intestinal Dysfunction by Maintaining Intestinal Barrier Integrity and Modulating the Microbiota in Septic Mice

**DOI:** 10.1155/2022/5026103

**Published:** 2022-05-29

**Authors:** Mina Zhang, Bo Lian, Rui Zhang, Yuhong Guo, Jingxia Zhao, Shasha He, Yunjing Bai, Ning Wang, Yan Lin, Xuerui Wang, Qingquan Liu, Xiaolong Xu

**Affiliations:** ^1^Beijing Hospital of Traditional Chinese Medicine, Capital Medical University, Beijing Institute of Traditional Chinese Medicine, Beijing 100010, China; ^2^Beijing University of Chinese Medicine, Beijing 100029, China; ^3^Beijing Key Laboratory of Basic Research with Traditional Chinese Medicine on Infectious Diseases, Beijing 100010, China

## Abstract

Sepsis-induced inflammatory response leads to intestinal damage and secondary bacterial translocation, causing systemic infections and eventually death. Emodin is a natural anthraquinone derivative in many plants with promising bioactivities. However, the effects and mechanisms of emodin on sepsis-induced intestinal dysfunctions have not been well clarified yet. We found that emodin treatment suppressed the inflammatory response in the intestines of septic mice. Intestinal barrier function was also improved by emodin through enhancing ZO-1 and occludin expression, which prevented the secondary translocation of *Escherichia coli.* By proteome microarray investigation, JNK2 was identified as a direct target of emodin. *In vitro* study also showed that emodin inhibited LPS-induced inflammatory response in intestinal epithelial cells. Nuclear factors including NF-*κ*B and AP-1 were further identified as downstream effectors of JNK2. Bioinformatic analysis based on 16s rRNA gene sequencing illustrated that emodin treatment significantly increased the alpha- and beta-diversity of gut microbiota in septic mice. Moreover, data according to functional prediction showed that emodin decreased the abundance of potential pathogenic bacteria in gut. Our findings have shown that emodin treatment prevented inflammatory induced barrier dysfunction and decreased the potential pathogenicity of lumen bacteria, reducing the hazard of lumen bacterial translocation during sepsis.

## 1. Introduction

Sepsis is a life-threatening disease caused by infection, concurrent immune system dysfunction, and multiorgan failure [1, 2]. According to the published data, sepsis remains to be the major cause of death in the intensive care unit (ICU) [3, 4]. Hospital mortality rates were 22.4% in the whole population, and the mortality rate associated with septic shock is even greater than 40% [5, 6]. Although therapeutic approaches for treating sepsis are continuously improving, mounting evidence shows an increased incidence of sepsis over the last 20 years [7].

Clinical evaluation of organ functioning was done by routine biomarkers, and most of the affected organs during sepsis and septic shock included the kidney, lung, and cardiovascular system [8]. However, the initial pathological mechanism and the intestinal tissue are also considered essential for sepsis [9–11]. The epithelium has been described as a motor for inflammatory response due to the secretion of proinflammatory cytokines and reactive oxygen species in response to pathogens [12]. Excessive inflammatory response in the intestine leads to damage of intestinal epithelial barrier and bacterial translocation, causing secondary infection in the local as well as the distant regions [13, 14]. Therefore, therapies that focus on the intestine might be helpful for the treatment of sepsis [15–17].

Emodin (1,3,8-trihydroxy-6-methylanthraquinone) is a natural anthraquinone derivative that exists in different kinds of Chinese herbs, such as *Rheum palmatum* L., *Cassia occidentalis*, *Polygonum multiflorum Thunb*, and *Aloe vera*. Several studies have reported that emodin possesses a wide spectrum of pharmacological effects, such as immunosuppressive, antibacterial, antiviral, antitumor, antiallergic, and antidiabetic properties [18, 19]. Recent evidence has reported that emodin treatment can be beneficial to sepsis treatment. Yu et al. have found that emodin significantly suppressed the overactivation of peripheral blood mononuclear cell isolation from severe septic patients [20]. Guo et al. have reported that emodin attenuates acute lung injury in septic rats via inhibition of excessive inflammation and apoptosis [21]. Chen et al. have suggested that emodin alleviates jejunal injury in rats with sepsis [22].

In the past decades, many strategies have been developed to excavate and investigate the potential inhibitors of proteins. For example, HuProt™20K microarray chip is a solid-phase protein array chip with nearly 20, 000 full-length proteins on the chip, providing an unbiased high-throughput screening method to discover targets of molecules [23, 24]. Molecular dynamics simulations of proteins, which have been used for 25 years ago, are by now widely used as strategies to investigate structure and dynamics under a variety of conditions [25–27]. By using these tools, researchers are capable of identifying potential combinations of proteins and molecules and understanding details of interactions.

Despite of accumulating interests on pharmacological and biological activities of emodin, there are still limited documents that reported how emodin decreases the pathogenicity of intestinal bacteria and prevents potential translocation, and this might in turn helps us to improve our understanding with regard to the pharmacological actions of emodin.

## 2. Materials and Methods

### 2.1. Reagents

Emodin (> 98% high-performance liquid chromatography (HPLC) purity) was purchased from Tauto Biotech (Shanghai, China). LPS (*Escherichia coli* O55:B5), DMSO, EMB, FITC, DAPI, hematoxylin and eosin, and goat antirabbit antibody were purchased from Sigma-Aldrich Chemical (St. Louis, MO, USA). Bicinchoninic acid (BCA) protein assay kit was purchased from Pierce (Rockford, IL, USA). The ELISA kits for TARC, IL-1*β*, and TNF-*α* were purchased from Cusabio (Wuhan, Hubei, China). The antibodies for ZO-1, Occludin, Claudin-1, cleaved Caspase-3, SIRT-1, JNK2, p-JNK2 pAP-1, pp65, and Actin were purchased from Abcam (Danvers, MA, USA).

### 2.2. Establishment of CLP Model

One hundred and fifty male BALB/c mice, 8 weeks old (18–22 g), were randomly divided into five treatment groups. Pentobarbital sodium (40 mg/kg·bw) was used as pain medicine during the surgery. Murine CLP model was established following protocol as described before [28]. Briefly, mice subjected to CLP surgery were fasted for 24 h with water permitted before operation. Anesthetized mice were then opened in abdominal cavity and ligated in the mid position, which comprised 50% of the cecum. A 5-gauge needle was used to puncture in the cecum (1 cm from ileocecal junction). Then, a small amount of feces was extruded from the puncture holes. Finally, cecum was cautiously relocated into the abdomen, and incision was sewed up. Mice were injected with 1 ml 37°C normal saline subcutaneously and fasted for another 12 h. In our hands, the 48-h mortality of this model is about 80%.

### 2.3. Cell Culture

MODE-K cells were purchased from the American Type Culture Collection (Rockville, MD, USA) and cultured in DMEM medium supplemented with 10% FBS and antibiotics (100 U/ml streptomycin and 100 U/ml penicillin) at 37°C in a humidified incubator with 5% CO_2_.

### 2.4. Transient Transfections with Constitutive Active JNK2

Plasmid expressing flag-tagged constitutively active JNK2 (pCDNA3 Flag MKK7B2Jnk2a2) is described before [29] and was purchased from Addgene (Cambridge, MA, USA). MODE-K cell was transfected by JNK2 (pCDNA3 Flag MKK7B2Jnk2a2) plasmid together with Lipofectamine 2000 reagent (Thermo Fisher CN, Shanghai, China) for an overnight transfection period. Conditioned medium was collected, and cells were then lysed. Western blotting was further employed to confirm the expression of JNK2.

### 2.5. Molecular Docking by AutoDock

To further characterize the interaction between Emodin and JNK2 protein, we conducted a molecular docking and simulation study using the crystal structure of JNK2 (RCSB PDB:3NPC) obtained from the Protein Data Bank (PDB) at https://www.rcsb.org/structure/3NPC. The Mol2 file of emodin was drawn and converted by ChemDraw. AutoDock software was used to flexibly dock JNK2 and Emodin and to get a preliminary docking phase structure. At least 50 docking results were generated, and the phase with the best docking energy was selected for structure extraction.

### 2.6. Molecular Dynamics Simulation by AMBER16

The optimal docking conformation was selected and simulated by molecular dynamics using AMBER16 software. The whole protein system was supported by gaff and ff14SB force fields. Hydrogen atoms and antagonistic ions are automatically added to make the system electrically neutral. The work flow of molecular dynamics simulation includes four steps: minimization, heating, equilibration, and production run. Two-step energy minimization was used and the dynamic simulation time was 100 ns. The average conformational structure of protein-molecule complex in 60-100 ns was used for subsequent analysis.

### 2.7. Enzyme-Linked Immunosorbent Assay (ELISA)

Briefly, we employed ELISA to detect the levels of the released cytokines *in vivo* and *in vitro*, including TARC, IL-1*β*, and TNF-*α*. Serum or cell supernatants were collected and immediately assayed. All protocols were performed as per the manufacturer's instructions.

### 2.8. Western Blotting

Proteins of samples were extracted by using lysis buffer and were quantified using a BCA assay kit. Proteins were separated by SDS-PAGE and electro-transferred to nitrocellulose membranes (Thermo Fisher CN, Shanghai, China), and then incubated with specific antibodies, including ZO-1, Occludin, Claudin-1, cleaved Caspase-3, SIRT-1, JNK2, p-JNK2 pAP-1, pp65, and Actin. Horseradish peroxidase (HRP-)-conjugated goat antirabbit IgG (1: 5000, Sigma-Aldrich CN, Shanghai, China) was employed as a secondary antibody.

### 2.9. Hematoxylin and Eosin (H&E) Staining

The ileum segments of each group were first immersed in 10% neutral buffered formaldehyde (formaldehyde 100 ml, distilled water 900 ml, and sodium dihydrogen phosphate 6.5 g) at room temperature for 48 h. The fixed specimens were then embedded in liquid paraffin and sliced into 5 *μ*m thickness. Sections were stained with hematoxylin and eosin, and the intestinal morphological changes were observed using a light microscope (Zeiss, Jena, TH, Germany). The degree of intestinal injury was scored on a scale from 0 to 5 using the criteria of Chiu's score method. A minimum of five randomly chosen fields from each rat were evaluated and averaged to determine mucosal damage.

### 2.10. Scanning Electron Microscopy (SEM)

The specimens of ileum chamber were fixed in 4% glutaraldehyde overnight and then post-fixed in cold 1% osmium tetroxide for 1 h, followed by three cacodylate buffer washes. After stepwise dehydration through a series of graded ethanol solutions, the samples were embedded in Epon-Araldite (EPON 812, Emicron, Shanghai, China). Ultrathin sections were stained with saturated uranyl acetate in 50% ethanol and lead citrate. Micromorphological pictures were obtained by employing scanning electron microscopy (S3400N, Hitachi, Ltd., Tokyo, Japan).

### 2.11. Intestinal Epithelial Paracellular Permeability Assay

The intestinal permeability of mice was measured using the flux of FITC-labeled dextran of molecular mass 4 KDa (FD4, Sigma-Aldrich) as previously described [21]. 44 mg/100 g body weight FITC-labeled 4-kDa dextran was orally administrated to each mouse. Blood was collected and centrifuged to isolate serum 5 h after FD-4 administration. The serum fluorescence intensity was assessed by a fluorometer (excitation, 492 nm; emission, 520 nm; BioTek).

### 2.12. Detection of JC-1 Fluorescence

Cells were harvested and stained by using the J-aggregate-forming lipophilic cation 5,5′,6,6′-tetraethylbenzimidazolocarbocyanine iodide (JC-1) dye (Thermo Fisher CN, Shanghai, China), which indicates the mitochondrial polarization by shifting its fluorescence emission from green (em 530 nm) to red (em 590 nm). Images were obtained using a ZEISS Axio Imager M2 fluorescence microscope and analyzed using AxioCam and Axiovision software.

### 2.13. 16S rRNA Gene Sequence Analysis of Intestinal Microbiota

Total genomic DNA of each fecal sample was extracted, and target sequences (V3-V4 region of 16S rDNA) were amplified by PCR. The products were then purified, quantified, and homogenized to get a sequencing library. Library quality control was performed for constructing libraries, and qualified libraries were sequenced on Illumina HiSeq 2500 (Illumina, San Diego, CA, USA). The original image data files obtained by high-throughput sequencing were converted into sequenced reads by base-calling analysis. The results were stored in FASTQ format file for further bioinformatics analysis.

### 2.14. Cytokine Microarray Analysis

Ileum samples or cell supernatants of each treatment group were assayed using a RayBio® mouse inflammation antibody array (RayBiotech AAM-INF-1, Norcross, GA, USA). Briefly, samples were diluted and incubated overnight in antibody array pools. The array slides were washed and incubated in a biotin-conjugated anticytokine mix for 2 h. Cy3-conjugated streptavidin was then added into the antibody array pools and incubated for another 2 h. Finally, the intensity of fluorescence signals was detected by using an InnoScan 300 Microarray Scanner (Innopsys, Carbonne, Occitanie, France).

### 2.15. Phosphoproteomics Analysis

Samples were first lysed in RIPA lysis buffer (Applygen Technologies, Beijing, China) with phosphatase inhibitor. Concentration of protein was measured using a BCA kit. 20 *μ*g of proteins of each sample was concentrated by SpeedVac (Thermo Fisher CN, Shanghai, China) for phosphopeptide enrichment and incubated with titanium dioxide beads (TiO2) for 1 h at room temperature and then pelleted by centrifugation. After washing, these phosphopeptides were eluted and concentrated in SpeedVac. All samples were desalted by C18 and loaded into a Nano-LC system. Peptides were separated, and MS/MS was conducted by using data-dependent acquisition. MS raw phosphoproteome data was analyzed by MaxQuant software. ANOVA test was performed to identify proteins that were significantly changed at phosphorylation level.

### 2.16. Protein Microarray Data Processing

Biotin–emodin was synthesized as described before [30]. Proteome microarrays were first treated with blocking buffer (1% BSA in 0.1% Tween 20 TBST) for 1 h at room temperature. Biotin–emodin and biotin were added to 10 *μ*M in blocking buffer and incubated on the proteome microarray for another 1 h. After that, microarray was incubated with Cy3-Streptavidin dilution (1 : 1000, Sigma-Aldrich CN, Shanghai, China) for 1 h and was spun dry at 250 × g for 3 min. A GenePix 4200A microarray scanner (Molecular Devices, San Jose, CA, USA) was used to visualize and record the results. An R script was applied to process the protein microarray data. The signal-to-noise ratio (SNR) was defined as the ratio of the median of foreground signal to the median of background signal. Another index, “Ratio,” was defined as SNR (biotin-emodin)/SNR (Biotin) [24].

### 2.17. Statistical Analysis

All data are presented as mean ± standard deviation (SD). Statistical analysis was performed using the GraphPad Prism 7 program (GraphPad, La Jolla, CA, USA). Log-rank test was used for Kaplan-Meier survival analysis. One-way analysis of variance (ANOVA) analysis was used to compare the statistical differences of data between two or more groups, followed by Tukey's test. *P* < 0.05 was considered statistically significant.

## 3. Results

### 3.1. Emodin Attenuates Intestinal Injury in Septic Mice

**Figure 1:**
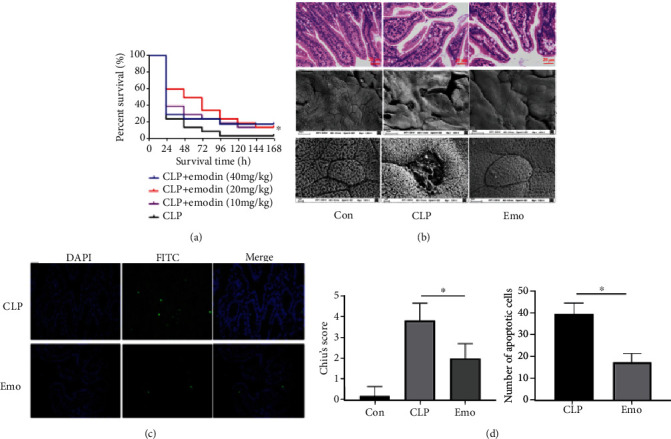
Emodin attenuates the intestinal injury of septic mice. (a) Emodin improves the survival rate of septic mice. Thirty mice of each treatment group were used for Kaplan-Meier investigation. The observation of mortality continued for 7 days, ∗*p* < 0.05 indicates significant difference compared with CLP surgery group. (b) Emodin preserves the intestinal structure of septic mice. HE staining and scanning electron microscope were employed for histopathological study. (c) Emodin suppresses the apoptosis in ileum of septic mice. The effect of emodin on apoptosis was estimated via TUNEL staining. Three fields of view were randomly selected (×400). The number of apoptotic cells was calculated in each field. (d) Quantification of intestinal injury via Chiu's score and count of apoptotic cells.

Cecal ligation and puncture (CLP) surgery was used to evaluate the therapeutic effects of emodin on septic mice. The Kaplan-Meier survival rate of each group revealed that oral administration of emodin 20 mg/kg·bw significantly decreased the mortality rate of septic mice through a 7-day observation period (Figure 1(a)). The images illustrated that CLP surgery not only caused erosion of upper villous surfaces but also widely destructed intestinal villi. Emodin treatment preserved the intestinal structure and attenuated the histopathological changes in septic mice (Figure 1(b)). Consisted with the histological changes of intestine, the Chiu's score in emodin-treated group was significantly lower than that in CLP group (Figure 1(d)). TUNEL assay results showed that emodin alleviated the incidence of apoptosis in the intestinal tissues to a certain extent (Figures 1(c) and 1(d)).

### 3.2. Emodin Inhibits Inflammatory Response in the Intestine of Septic Mice

**Figure 2:**
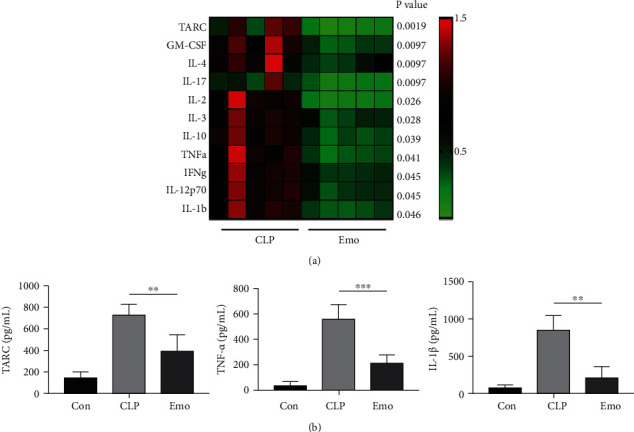
Emodin inhibits inflammatory response in intestine of septic mice. (a) Cytokines microarray was employed for detection of inflammatory factors. Fold changes of CLP group were indicated in the heat map, and differences between mean values were assessed. *p* < 0.05 indicates significant difference compared with CLP group. (b) Identification of results from cytokines microarray by ELISA. Data represent the mean ± SD of three independent experiments, and differences between mean values were assessed by one-way ANOVA. ∗∗*p* < 0.01, ∗∗∗*p* < 0.001 indicate significant differences compared with CLP group.

Cytokine antibody array was performed to evaluate the anti-inflammatory effects of emodin on the intestines of septic mice (in a total of 40 cytokines are listed in Table S1). The results revealed that 11 highly expressed cytokines in the ileum of CLP mice were significantly downregulated in the presence of emodin, including thymus and activation-regulated chemokine (TARC), granulocyte macrophage colony-stimulating factor (GM-CSF), interleukin (IL)-1*β*, IL-4, IL-17, IL-2, IL-3, IL-10, IL-12p70, tumor necrosis factor (TNF)-*α*, and interferon (IFN)-*γ* (Figure 2(a)). Considering the false positives by high-throughput examination, further verification of our findings was done by testing some of these cytokines through ELISA. These data showed consistent results, as the expression of TARC, TNF-*α*, and IL-1*β* in the ileum was remarkably decreased by emodin treatment (Figure 2(b)).

### 3.3. Emodin Protects against Sepsis-Induced Intestinal Barrier Dysfunction

**Figure 3:**
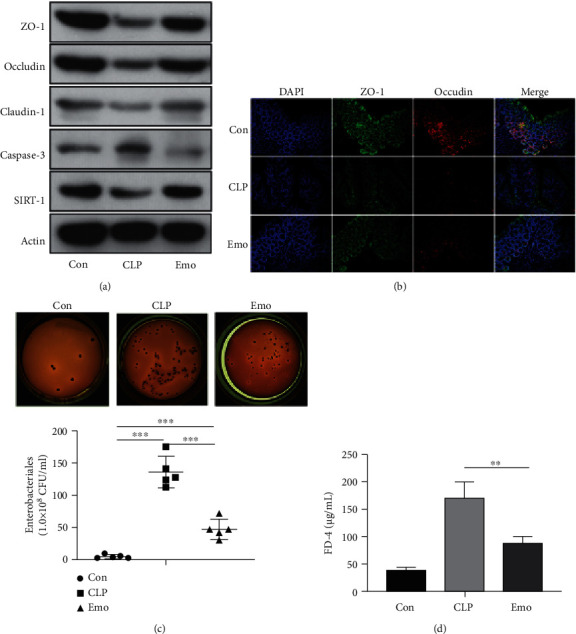
Emodin restored the intestinal barrier function of septic mice. (a) Regulatory effect of emodin on ZO-1, occludin, claudin-1, cleaved caspase-3, and SIRT-1. (b) Immunofluorescence on ZO-1 and occludin by using confocal microscopy. (c) Determination of *E. coli* transplanted colonies in MLNs. Statistics of bacterial colonies in the mesenteric lymph nodes of mice in each group by counting CFU (colony-forming unit). ∗*p* < 0.05, ∗∗*p* < 0.001, and ∗∗∗*p* < 0.001 with comparisons indicated by lines (*n* = 5 per group). (d) Effects of emodin on intestinal permeability in CLP mice. Intestinal permeability was determined by detecting plasma FD4. Data are presented as means ± SEM from three independent experiments, and differences between mean values were assessed by one-way ANOVA.

The functioning of important proteins in the intestinal barrier was evaluated. The results of western blotting showed that the expressions of zonula occludens-1 (ZO-1), occludin, and claudin-1 in the intestines of septic mice were significantly lowered than that in control group. Emodin treatment restored the expressions of ZO-1 and occludin, but had limited regulatory effects on claudin-1. Moreover, several apoptosis-associated proteins including caspase-3 and Sirtuin 1 (SIRT1) were detected. The results indicated that emodin notably inhibited the expression of cleaved caspase-3 in septic mice. In Figure 3(a), the expression of SIRT-1 is found to be partially restored by emodin treatment (gray value comparison in Figure S1). The location and expression of ZO-1 and occludin were further investigated via immunofluorescence. These data illustrated that emodin recovered the impaired expressions of ZO-1 and occludin in septic mice (Figure 3(b)). Oxidative stress indicators including ROS and NO were decreased by emodin treatment (Figure S2). Bacterial translocation is an important outcome of intestinal barrier dysfunction. The amount of *Escherichia coli* (*E. coli*) in mesenteric lymph nodes (MLNs) of each group was assessed. The results showed that the number of *E. coli* in MLNs of septic mice was dramatically higher than that in control group. Emodin treatment significantly decreased the amount of *E. coli* (Figure 3(c)). Intestinal permeability was evaluated *in vivo* using FD4. Mice in the emodin treatment group had significantly lower levels of plasma FD4, as compared with the CLP group (Figure 3(d)).

### 3.4. JNK2 Is Identified as a Direct Target of Emodin

**Figure 4:**
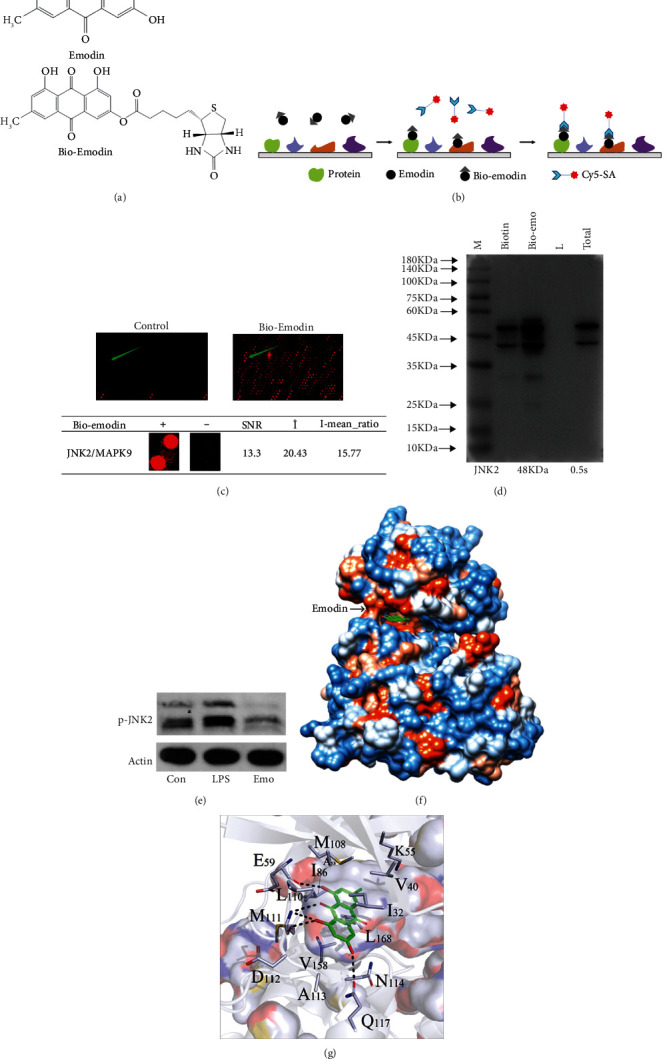
Identification of Emodin-binding proteins. (a) Chemical structure of emodin and bio-emodin (fluorescent tag). (b) A schematic chart of identification of bio-emodin-binding protein using a proteome microarray. (c) Images of location of JNK2 in the biotin control and the bio-emodin microarray and details of the bonding. (d) Pulldown detection for the interaction of emodin and JNK2 in MODE-K cells. M: marker; L: loading buffer. (e) Effect of emodin on JNK2 phosphorylation. Western blot was employed to detect p-JNK2 expression, and three independent experiments were performed. (f) Emodin and JNK2 are shown as cartoon and sticks, respectively. It is color coded according to the electrostatic potential. Blue: hydrophilic amino acid; white: neutral amino acid; and orange: hydrophobic amino acid. (g) Electrostatic interaction pattern diagram of pockets around small molecules. Details of interaction is shown color-coded according to the electrostatic potential.

To explore the binding proteins of emodin, bio-emodin (Figure 4(a)) was synthesized, and a proteomic microarray containing 16,368 proteins with binding affinity was employed to purify the N-terminal GST-tagged proteins. Bio-emodin or biotin was probed on proteome microarray and was further incubated with Cy3-conjugated streptavidin (Cy3-SA), causing protein/bio-emodin interactions (Figure 4(b)). The location of JNK2 microarray was indicated, and the fluorescence signal values of control and bio-emodin microarrays were compared (Figure 4(c)). The signal to noise ratio (SNR) for each spot demonstrated the binding affinity of emodin on proteins. The SNR of emodin/JNK2 was shown to be 13.3, indicating a strong interaction (Figure 4(c)). SPR detection also illustrated that there was a strong combination between emodin and JNK2, with affinity of 1.19e-5 M (Figure S3). The data from pulldown detection further confirmed the effects of emodin/JNK2 combination (Figure 4(d)). The phosphorylation level of JNK2 was inhibited by emodin treatment (Figure 4(e)). During docking, at least 50 docking results were generated, and the phase with the best docking energy was selected for structure extraction, and AMBER16 software was used for molecular dynamics simulation. According to the simulation data, the Emodin-JNK2 combination stabilized at 1.8 Å, with RMSD value of -8.05 kcal/mol (Figure S4). Docking data further showed that from the surface map of the hydrophobicity of the protein, it was revealed that the surface of the protein was dominated by polar amino acids. The protein surface is mainly composed of polar amino acids. The small molecule binding pocket is a hydrophobic binding pocket region composed of pure hydrophobic amino acids. Moreover, the physical and chemical properties of planar rings on small molecules match the binding cavity (Figure 4(f)). The analysis of molecular interaction between JNK2 and emodin showed that the sugar ring group consisted of surrounding amino acids with K55 and Q117 polar amino acids, among which the Q117 could form a strong hydrogen bond with that of hydroxyl -OH group at the top of emodin. Benzene ring group comprises of surrounding amino acids of hydrophobic amino acids such as L168, I32, V158, M111, I86, and L110 and polar amino acids such as E109 and N114 (Figure 4(g)).

### 3.5. JNK2 Is Essential for Emodin to Exhibit Activities

**Figure 5:**
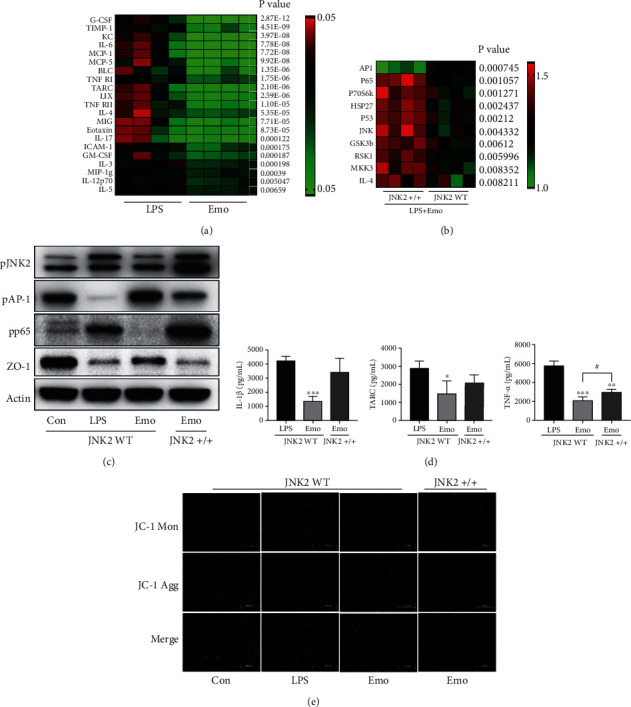
Role of JNK2 in emodin exhibited activities. (a) Cytokine microarray was employed for the detection of inflammatory cytokines. Fold changes of LPS group were indicated in the heat map, and differences between mean values were assessed. *p* < 0.05 indicates significant difference compared with LPS group. (b) Proteins' phosphorylation was examined using a customized phosphorylation microarray. Fold changes of LPS group were indicated in the heat map, and differences between mean values were assessed by modulated *t*-statistics. *p* < 0.05 indicates significant difference compared between WT and JNK2 overexpressed cells. (c) WT cells or JNK2 overexpression cells were challenged, and samples of each group were obtained to detect the expression of pJNK2, pAP-1, pp65, and ZO-1 by western blot. (d) WT cells or JNK2 overexpression cells were challenged, and samples of each group were obtained for ELISA detection. Data represent the mean ± SD of three independent experiments, and differences between mean values were assessed by one-way ANOVA. ∗*p* < 0.05, ∗∗*p* < 0.01, ∗∗∗*p* < 0.001, and #*p* < 0.05 indicate significant differences compared with LPS group. (e) Detection of mitochondrial membrane potential by JC-1 staining. Cells were treated as indicated. The double staining of cells by JC-1 is visible either as green for JC1 monomers (Mon) or red for J-aggregates (Agg).

To better understand on how emodin/JNK2 combination affects the process of intestinal injury in septic mice, MODE-K cell line was used to reveal the underlying mechanisms. The effects of emodin on inflammatory response were estimated using a 40-cytokine microarray. Similar to the results of *in vivo* study, emodin treatment suppressed the overexpression of 21 cytokines in LPS-challenged cells (Figure 5(a)). Furthermore, we found that emodin treatment downregulated the phosphorylation levels of JNK2, p65, P70S6K, and p53 but enhanced p-AP1 expression in the presence of LPS, by using phosphorylation microarray (in a total of 17 proteins are listed in Table S2) and western blot detection (Figures 5(b) and 5(c)). However, enhancing the expression of JNK2 partially reversed the regulatory effects of emodin on the phosphorylation of pp65 and p-AP1. Also, emodin treatment that induced the expression of ZO-1 was prevented by JNK2 overexpression (Figure 5(c)), in accordance with the gray value comparison (Figure S5). The anti-inflammatory effects of emodin were also prohibited in JNK2 overexpression cells (Figure 5(d)). Moreover, emodin treatment reversed the reduction of mitochondrial membrane potential caused by LPS, and this effect was dismissed by JNK2 overexpression (Figure 5(e)).

### 3.6. Emodin Regulates Intestinal Community Structure in Septic Mice

**Figure 6:**
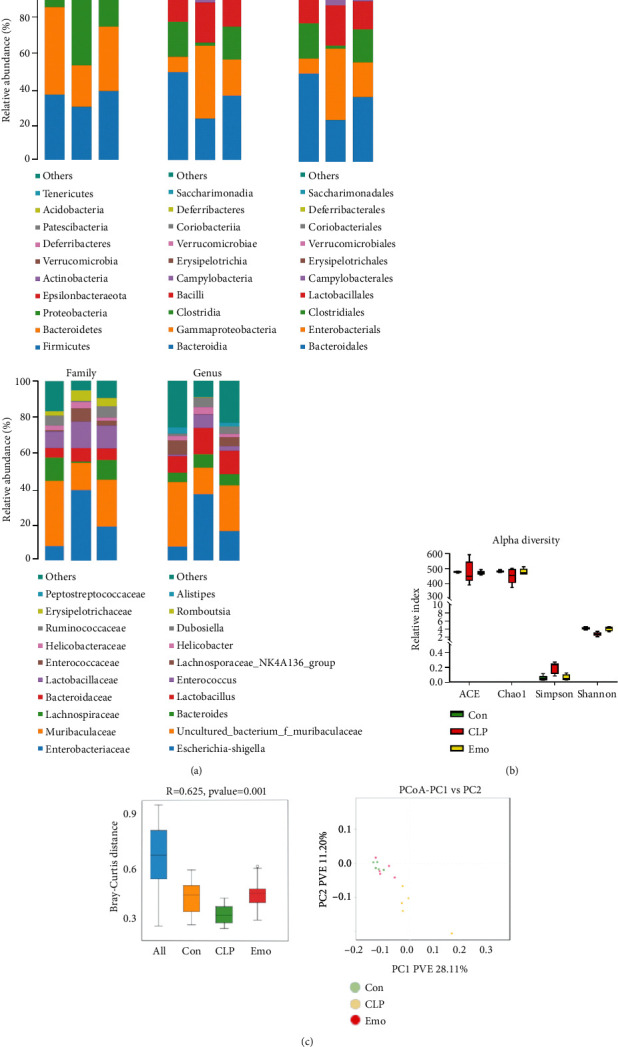
Emodin regulates intestinal community structure in septic mice. (a) Histogram of species distribution. The chart was the pie chart of the distribution of species at each level: including phyla, class, order, family, and genus. One color represented one specie; the length of the color block (histogram) or area of the color block (pie chart) presented the relative richness proportion of the species. Only species with top ten richness level were shown; the rest species were combined as others in the chart. (b) Alpha diversity of each treatment group. Use alpha diversity analysis to study the species diversity within a single sample. Ace, Chao1, Shannon, and Simpson indexes of each sample were statistically calculated at 97% similarity level. (c) Beta diversity of each treatment group. Beta diversity analysis was used to compare the differences of species diversity between different samples, using Bray-Curtis based PCoA and anosim analysis.

So far, the effects of emodin on intestinal microbiome of septic mice have not been reported yet. By using 16s rRNA gene sequencing, we noticed significant changes in the intestinal community structure and species distribution of septic mice at different level, including phyla, class, order, family, and genus levels. And these trends of shifting were partially reversed by emodin treatment (Figure 6(a)). Moreover, species abundance and diversity of intestinal microbiome were shown to be significantly decreased in septic mice. In the alpha diversity analysis, the data illustrated that the Shannon and Simpson indexes showed notable differences between the control group and the sepsis group and could be restored by emodin treatment. However, no meaningful results were obtained by Ace and Chao indexes (Figure 6(b)). In the beta diversity analysis, emodin treatment also reversed the changes caused by sepsis according to the results of PCoA and Anosim analysis (Figure 6(c)).

### 3.7. Potential Functional Analysis on the Shift of Microbiota in Septic Mice

**Figure 7:**
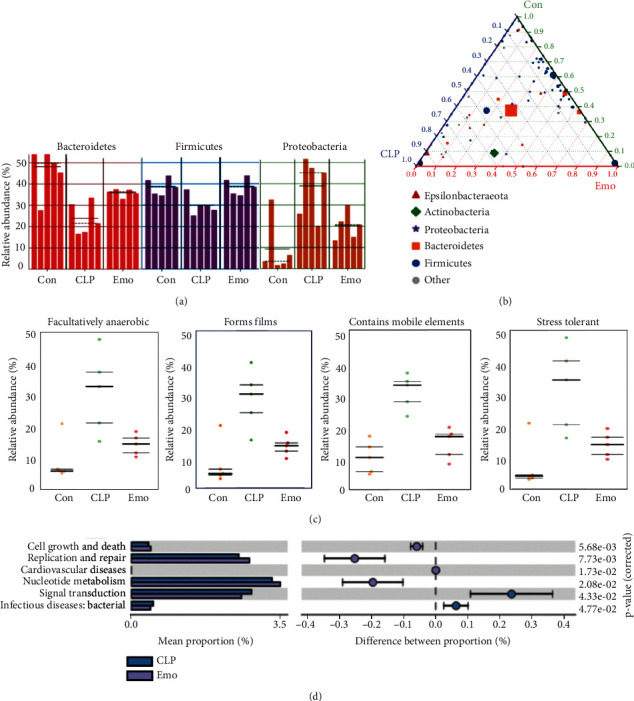
Functional analysis on the shift of microbiota in septic mice. (a) LEfSe analysis of samples between groups. The histogram of LDA value distribution and evolutionary branch graph of LEfSe analysis is illustrated. (b) Ternary diagram of samples between groups. The ternary diagram is drawn by using Python. The proportion and relationship of different species in the samples is shown through the triangle diagram. Green: control group; blue: CLP group; and red: Emo group. (c) BugBase function prediction. *x*-axis represents the group name, and *y*-axis represents the relative abundance percentage. The three lines are the lower quartile, the mean, and the upper quartile, respectively, from the bottom up. (d) COG function prediction. The analysis results are shown, reflecting the function distribution and abundance of the sequences in samples.

According to LEfSe and ternary diagram analysis, we found that the abundance of *Proteobacteria* was decreased while that of *Firmicutes* and *Bacteroidetes* was increased by emodin when compared to septic mice (Figures 7(a) and 7(b)). Further functional prediction based on BugBase was employed to predict the microbial phenotypes and potential features. The results illustrated that the abundance of facultatively anaerobic bacteria with the ability of resistance towards host immune systems was shown to be significantly increased in septic mice and could be reduced by emodin (Figure 7(c)). The COG function prediction that reflects the functional distribution and abundance of the sequences in samples was also performed. It is noteworthy that the functional genes of microbial communities between each group involved in metabolic pathways included cell growth and death, replication and repair, and bacterial infectious diseases (Figure 7(d)).

## 4. Discussion

Sepsis is a life-threatening organ dysfunction that is caused by dysregulation of host response to infection [24]. In the early stage of sepsis, the excessive release of inflammatory cytokines caused by infections has led to multiorgan damage and ischemia reperfusion injury, for instance, intestinal barrier function disorder. It has been well documented that the gastrointestinal tract plays a key role in the pathophysiology of sepsis, and this is because the intestinal-derived infection is the most common complication in patients who suffer from trauma, burn, sepsis, and septic shock [31]. Under an overwhelming infectious condition, the inflammatory milieu contributes to intestinal hyperpermeability, subsequently resulting in the translocation of intact bacteria, endotoxin, and various kinds of metabolites from the lumen into previously sterile locations, exacerbating sepsis and perpetuating the inflammatory response and MODS [32]. Therefore, intestinal dysfunction also triggers the pathogenesis of sepsis and MODS [33].

Emodin is a naturally occurring anthraquinone that possesses well-established biological properties. Ho et al. have proved that emodin had promising antiviral activities by blocking the interaction between S protein and ACE2, thus suppressing the infectivity of S protein-pseudotyped retrovirus to cells [34]. Dong et al. have found that emodin has remarkable antibacterial effects on gram-positive and drug-resistant bacteria, such as *Staphylococcus aureus*, *Bacillus subtilis*, and methicillin-resistant *S. aureus* (MRSA) [18]. Besides the anti-infection effects, emodin treatment also had organ protection properties, such as neuroprotection, hepatoprotection, and antiallergic activities [35–37]. Recently, Chen et al. have reported that emodin alleviates jejunal injury in septic rats by inhibiting pJAK1/pSTAT3-mediated inflammatory response and Bcl2/Bax involved apoptosis, partially revealing the effects of emodin on regulating intestinal dysfunction during sepsis [22]. In this study, the role of emodin in protecting the intestinal barrier functions and to dig its underlying mechanism in treating sepsis was assessed. In accordance with the documented data [38], emodin has shown to have reproducible therapeutic effects in reducing the mortality of septic mice. Meanwhile, the protective effects of emodin on alleviating the intestinal injury caused by CLP surgery via using multidimensional analysis methods, including HE staining, scanning electron microscope, and TUNEL assay. Considering the importance of intestine in the pathological process of sepsis, the protective effects of emodin on intestinal structure should be considered as a promising preparation in the prognosis of sepsis.

The intestinal tract is lined up by a single layer of epithelial cells, which acts as a physical and metabolic barrier in the paracellular movement of water, solutes, and immune regulatory factors, while the intrusion of potentially intraluminal toxins, pathogens, and antigens was intercepted into the mesenteric and circulation of lymph nodes [39, 40]. The intestinal mucosa is protected and regulated by epithelial junctional complexes such as adherens junction and tight junctions (TJs). Several molecules that act as contributors of TJs such as occludin, transmembrane protein claudins, and ZO-1 have been identified [41]. To evaluate the effects of emodin on the modulation of TJs in septic mice, the expression levels of ZO-1, occludin, and claudin-1, whose defects might lead to the dysfunction of TJs, were investigated. Similar to the former published data, the expressions of ZO-1, occludin, and claudin-1 were impaired in the intestines of septic mice. Emodin treatment recovered the expression of ZO-1 and occludin, but showed no regulatory effects on claudin-1. In light of different expression patterns and upstream modulatory mechanisms between claudin-1 and ZO-1/occluding [42, 43], emodin treatment modulated the expression of ZO-1 and occludin via a distinct signaling pathway from claudin-1. As a consequence of intestinal barrier dysfunction, bacterial translocation is of great significance during the pathological process of sepsis [32]. Therefore, the effects of emodin on bacterial translocation in septic mice were estimated by counting the amount of *E. coli* in MLNs. Not surprisingly, the number of *E. coli* was dramatically increased in the MLNs of septic mice, indicating a potential situation for secondary infection. Emodin treatment restrained excessive growth of translocated bacteria caused by CLP surgery, and this might be due to the protective property of emodin on TJs and barrier function.

JNKs, including three distinct genes JNK1, JNK2, and JNK3, are a family of stress-activated serine threonine protein kinases of the MAPK. Unlike JNK3 that is specifically expressed in central nervous system, JNK1 and JNK2 have broad tissue distribution and play an important role in inflammatory response, apoptosis, and cell signaling [44, 45]. Alternative splicing in two different parts produce different JNK isoforms. Differences in protein kinases IX and X produce JNK1 and JNK2 *α* and *β* variants. The same type with a molecular weight of 46 kDa and 54 kDa is generated by an alternative splicing of the C-terminus [46]. By proteome microarray and bioinformatic analysis, JNK2 is shown to act as a potential target of emodin, with an SNR of 13.3. The results of pulldown detection also confirmed the interaction between emodin and JNK2. The HuProt™20K microarray chip that we used is a solid-phase protein array chip with nearly 20, 000 full-length proteins on the chip, covering 81% of the ORF region of the human genome. Mutations of JNK2 have been included in the HuProt™20K chip. According to the microarray result, we found that emodin binds to the JNK2 (PDB ID: 3NPC). Therefore, we used this crystal structure (3NPC) to investigate the interaction of Emodin-JNK2 complex. According to the simulated data, emodin treatment caused embedding of JNK2 into the active region mainly via hydrogen bonding and hydrophobic interaction, which are two critical binding forces for complex stabilization, and included many amino acids such as Q117, K55, L168, I32, M111, E109, V158, M111, I86, and L110. In light of the biological effects of JNK2 through phosphorylation modulation, the effects of emodin on JNK2 phosphorylation was further detected. As expected, emodin treatment prevented pJNK2 expression in the intestines of septic mice, which might in turn inhibit the activation of downstream regulators.

Due to direct binding of emodin and JNK2, we studied on how the interaction contributes in the protection of intestinal integrity during sepsis. MODE-K cell line was used to study the underlying mechanisms *in vitro*. In accordance with the *in vitro* findings, excessive inflammatory response and TJ dysfunction caused by LPS were shown to be attenuated. Practically, 21 of 40 inflammatory cytokines were decreased by emodin treatment by microarray detection. TJs-related proteins, including ZO-1 and occludin, were also restored in the presence of emodin. However, the effects of emodin in a JNK2 overexpression cell line were partially reversed, indicating the potential role of JNK2 in emodin exhibited activities. To better understand the regulatory mechanism of emodin, a customized phosphorylation microarray chip containing 17 proteins, whose phosphorylation are important for cellular signaling, has been established. By comparing the results between WT and JNK2 overexpression cell line, significant changes associated with pp65 and pAP-1 have drawn our attention. When compared with WT cells, the expression of pp65 was enhanced, but pAP-1 was decreased when JNK2 was overexpressed in the presence of LPS and emodin. P65 is a subunit of NF-*κ*B, which is a nuclear transcription factor that has been well documented as a regulator of immune function, proliferation, and migration [47]. Therefore, emodin has anti-inflammatory effects by binding to JNK2, which thereby prevents the activation of NF-*κ*B-mediated signaling pathways, contributing to the alleviation of inflammatory injury in the intestines. This study revealed another point with regard to the role of emodin/JNK2 to TJs and barrier function. Recently, Fabio et al. have reported that inhibition of JNK has enhanced epithelial barrier function through modulation of claudin [48]. Our findings have revealed potential regulatory mechanism of JNK2/AP1 in TJ functioning, which has not been reported yet. Based on our data, emodin/JNK2 complex inactivated JNK2, leading to the enhancement of AP1 expression. Overexpression of JNK2 has decreased AP1 expression. This is similar to the study conducted by Kanaga et al., which revealed that JNK2 is preferentially bound to c-Jun, thus contributing to c-Jun degradation [49]. Therefore, it is concluded that emodin binds to JNK2 and inhibits NF-*κ*B-mediated excessive inflammatory response, and emodin treatment inactivates JNK2 and contributes to the accumulation of AP-1, which is considered essential for restoring TJs and intestinal barrier function during sepsis.

The importance of intestinal microbiota is clearly evident in considering the risks of developing sepsis [31, 50, 51]. However, no data is available with regard to the modulatory property of emodin on the gut microbiota of septic mice. It has been reported that the lower microbial diversity is related to higher incidence of various diseases, including sepsis [52]. Our data from 16SrRNA also showed that both alpha and beta diversity were significantly decreased in septic mice, which could be partially restored by emodin treatment. Recent studies have reported that the intestinal microecology in critically ill patients showed significant alterations, which is characterized by augmentation of opportunistic *Proteobacteria* and reduction of commensals *Firmicutes* and *Bacteroidetes* [53, 54]. The role of *Proteobacteria*, including *Salmonella*, *E. coli*, and *Helicobacter pylori*, is very small in the health of animals as well as humans. However, its scale increases in patients with intestinal infections or colorectal cancer. In septic mice, the *Proteobacteria* had higher chances of community structure when compared with the control group. This might be due to impaired ability of eliminating pathogenic bacteria during sepsis. Emodin treatment notably decreased the amount of *Proteobacteria,* which might be important in protecting the host from opportunistic pathogens. *Firmicutes* and *Bacteroidetes* are two important bacterial phyla in the host that participate in the regulation of metabolism and maintain energy balance. Maintaining their abundance is of great value for the survival and prognosis of septic patients [55, 56]. Decreased abundance of *Firmicutes* and *Bacteroidetes* was observed in septic mice, which in turn was restored by emodin treatment. This indicated potential regulatory effects on the dysfunction of energy metabolism. Different from the strict anaerobes, which is considered essential for protecting against exogenous microorganisms and preventing bacterial translocation, facultative bacterial anaerobes such as *Enterobacteriaceae* and *Bacteroideae* facilitate the development of sepsis [57–59]. Emodin suppressed the abundance of facultative anaerobes, which is increased in septic mice. Also, emodin has potential inhibitory effects in forming the biofilms, accounting for over 80% of microbial infections in human body and creating difficulties for clinical treatment of sepsis because of high resistance to antimicrobial agents [60, 61]. Besides, we found that emodin decreases the abundance of bacteria that contain mobile elements and possess higher ability to resist external stress and host immune systems. The above data illustrated that apart from the modulation of inflammatory response and protection of barrier integrity, emodin regulated intestinal microbiota, benefitting the treatment of sepsis.

## 5. Conclusions

In summary, emodin directly binds to JNK2, on one hand, inhibiting NF-*κ*B induced excessive inflammatory damage and, on the other hand, maintaining the intestinal barrier integrity via promoting AP-1 involved TJ function in septic mice. In addition, emodin exhibits regulatory effects on intestinal microbiota which might be due to increased species diversity and reduced potential pathogenicity of lumen bacteria. This work illustrated pharmacological actions of emodin in protecting the intestinal epithelial barrier function, thus preventing secondary bacterial translocation and infection in septic mice, and providing more evidence for treating sepsis.

## Data Availability

The datasets used and/or analyzed during the current study are available from the corresponding author on reasonable request.

## Abbreviations

JNK2: C-Jun N-terminal kinase 2
NF-*κ*B: Nuclear factor-kappaB
AP-1: Activator protein-1
CLP: Cecal ligation and puncture
TARC: Thymus and activation-regulated chemokine
GM-CSF: Granulocyte macrophage colony-stimulating factor
IL: Interleukin
TNF: Tumor necrosis factor
IFN: Interferon
ZO-1: Zonula occludens-1
SIRT1: Sirtuin 1
MLNs: Mesenteric lymph nodes
Cy3-SA: Cy3-conjugated streptavidin
SNR: Signal to noise ratio
TJs: Tight junctions.

## Supplementary Materials

Supplementary Materials Table S1. Cytokines microarray with 40 factors. Table S2. Phosphorylation microarray with 17 proteins. Figure S1. Quantification of WB in Figure 3(a). Data represent the mean ± SD of three independent experiments, and differences between mean values were assessed by one-way ANOVA. ∗*p* < 0.05 and ∗∗*p* < 0.01 indicate significant differences compared with CLP group. Figure S2. Inhibitory effect of emodin on the oxidative stress in septic mice. Data represents the mean ± SD of three independent experiments, and differences between mean values were assessed by one-way ANOVA. ∗∗∗*p* < 0.001 indicates significant differences compared with control group. Figure S3. The SPR detection of JNK2 and Emodin combination. Figure S4. Root mean square deviation (RMSD) of the simulation. The abscissa represents the time and the ordinate represents the specific value of RMSD. Figure S5. Quantification of WB in Figure 5(c).
