# Supplementary material for: Emodin Ameliorates Intestinal Dysfunction by Maintaining Intestinal Barrier Integrity and Modulating the Microbiota in Septic Mice
Source: Mediators Inflamm. 2022 May 29;2022:5026103. doi: 10.1155/2022/5026103 (PMC9168211; doi:10.1155/2022/5026103)
Supplement: Supplementary Materials — Table S1. Cytokines microarray with 40 factors. Table S2. Phosphorylation microarray with 17 proteins. Figure S1. Quantification of WB in Figure 3(a). Data represent the mean ± SD of three independent experiments, and differences between mean values were assessed by one-way ANOVA. ∗p < 0.05 and ∗∗p < 0.01 indicate significant differences compared with CLP group. Figure S2. Inhibitory effect of emodin on the oxidative stress in septic mice. Data represents the mean ± SD of three independent experiments, and differences between mean values were assessed by one-way ANOVA. ∗∗∗p < 0.001 indicates significant differences compared with control group. Figure S3. The SPR detection of JNK2 and Emodin combination. Figure S4. Root mean square deviation (RMSD) of the simulation. The abscissa represents the time and the ordinate represents the specific value of RMSD. Figure S5. Quantification of WB in Figure 5(c). [file 5026103.f1.docx]

**Supplementary -1**

**Table S1. Cytokines microarray with 40 factors**

| Cytokine List | | | | | | | |
| --- | --- | --- | --- | --- | --- | --- | --- |
| BLC | CD30 | Eotaxin-1 | Eotaxin-2 | G-CSF | TIMP-1 | KC | MCP-1 |
| MCP-5 | FasL | TNF-R1 | TARC | LIX | TNF-RII | IL-4 | MIG |
| IL-17 | IL-1α | ICAM-1 | GM-CSF | IL-5 | MIP-1γ | G-CSF | CCL1 |
| IFN-γ | IL-10 | IL-12p70 | IL-13 | IL-15 | IL-2 | IL-21 | IL-7 |
| IL-6 | Leptin | M-CSF | MIP-1α | CXCL4 | CCL5 | TNFα | IL-1β |

**Table S2. Phosphorylation microarray with 17 proteins**

| Phosphorylation Protein List | | | | | | | |
| --- | --- | --- | --- | --- | --- | --- | --- |
| Akt | CREB | P65 | GSK3α | GSK3β | HSP27 | JNK | MEK |
| MKK3 | MKK6 | MSK2 | mTOR | AP1 | p53 | P70S6K | RSK1 |
| RSK2 |  |  |  |  |  |  |  |

**Figure S1.**


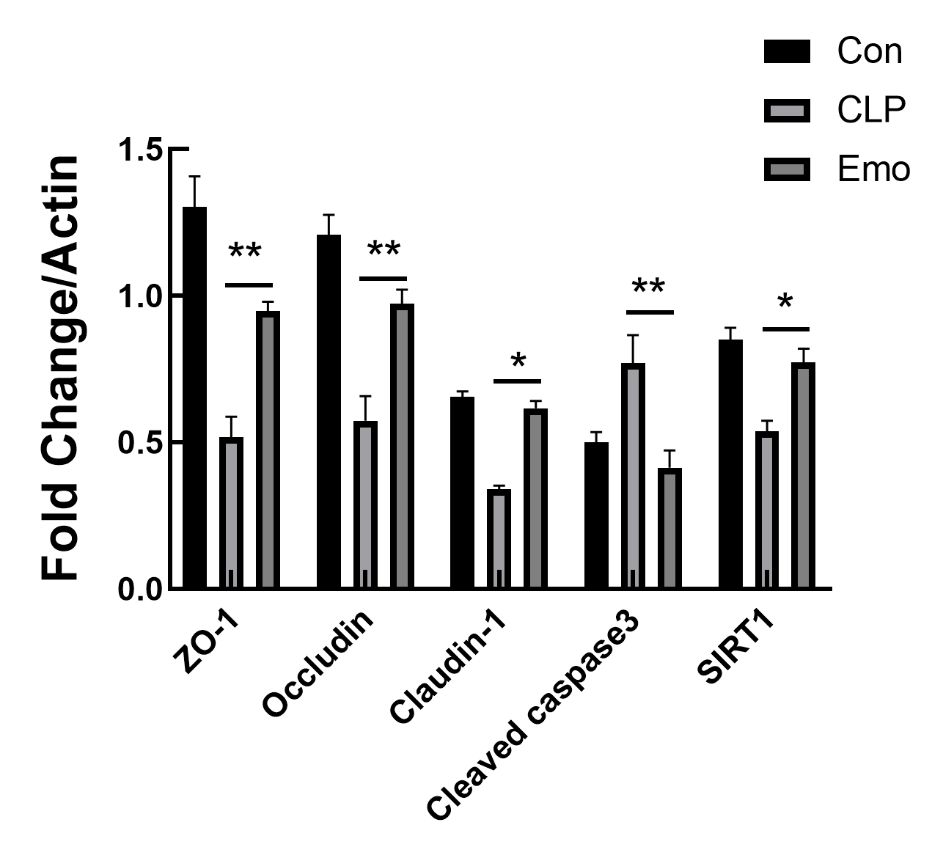


**Figure S1.** Quantification of WB in Figure 3A. Data represent the mean ± SD of three independent experiments and differences between mean values were assessed by one-way ANOVA. *p < 0.05, **p < 0.01 indicate significant differences compared with CLP group.

**Figure S2**

**
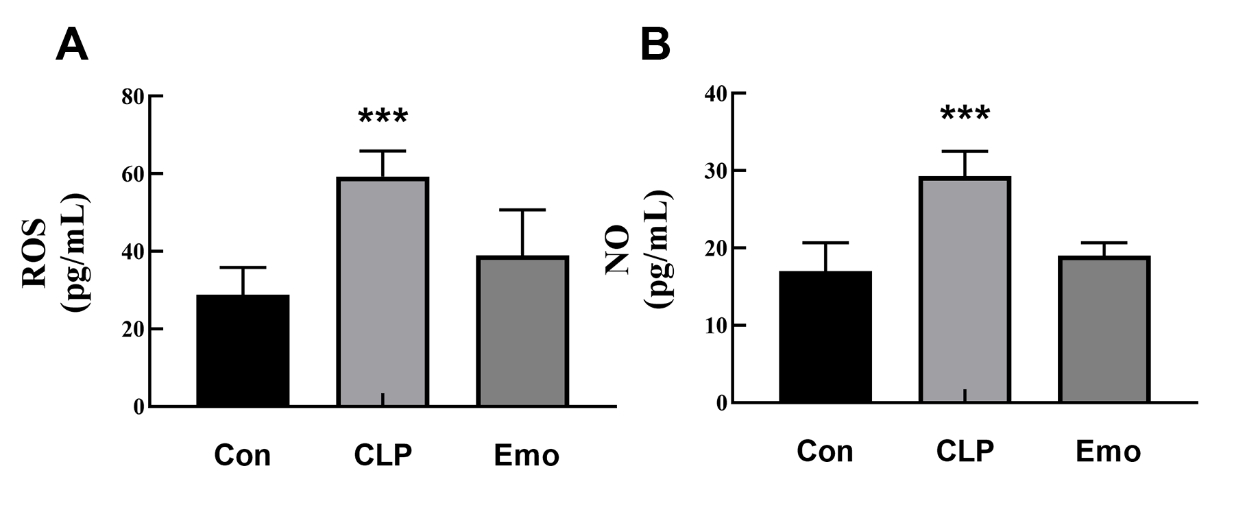
**

**Figure S2.** Inhibitory effect of emodin on the oxidative stress in septic mice. Data represents the mean ± SD of three independent experiments and differences between mean values were assessed by one-way ANOVA. ***p < 0.001 indicate significant differences compared with control group.

**Figure S3**





**Figure S3.** The SPR detection of JNK2 and Emodin combination.

**Figure S4**





**Figure S4.** Root mean square deviation (RMSD) of the simulation. The abscissa represents the time and the ordinate represents the specific value of RMSD.

**Figure S5. Quantification of WB in Figure 5C**


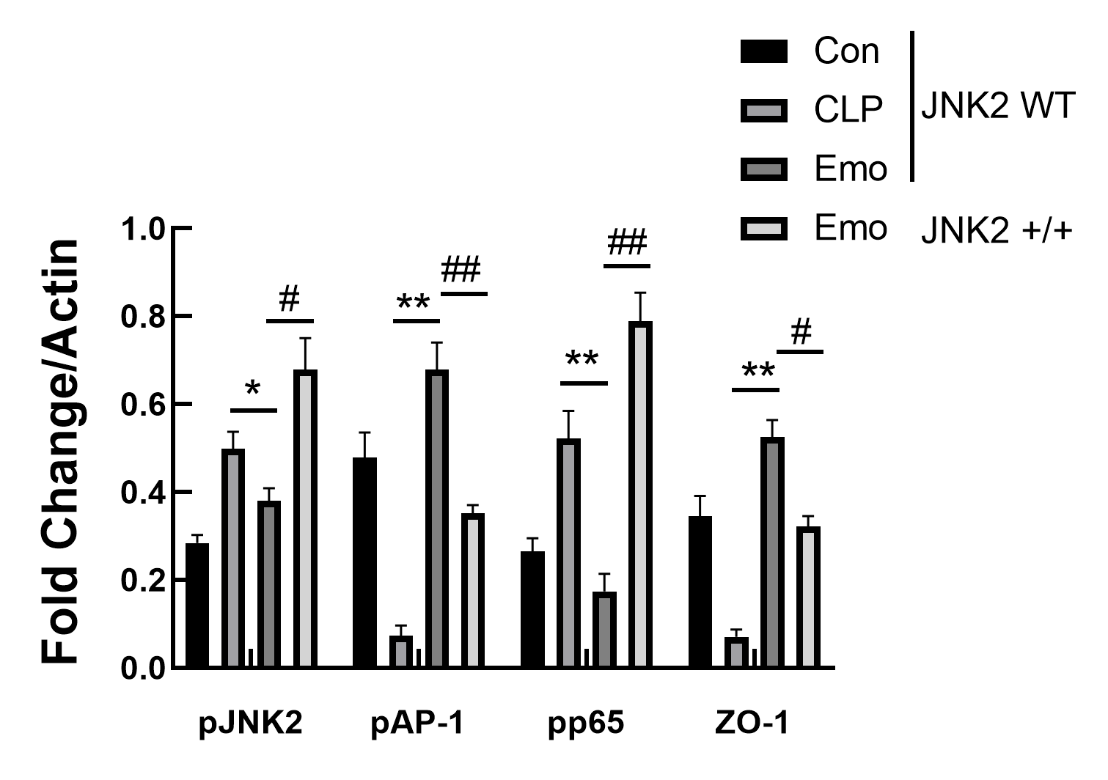


**Figure S5**. Data represent the mean ± SD of three independent experiments and differences between mean values were assessed by one-way ANOVA. *p < 0.05, **p < 0.01 indicate significant differences compared with CLP group. #p < 0.05, ##p < 0.01 indicate significant differences compared with JNK2 +/+ group.
